# FGF10 Protects Against Renal Ischemia/Reperfusion Injury by Regulating Autophagy and Inflammatory Signaling

**DOI:** 10.3389/fgene.2018.00556

**Published:** 2018-11-23

**Authors:** Xiaohua Tan, Hongmei Zhu, Qianyu Tao, Lisha Guo, Tianfang Jiang, Le Xu, Ruo Yang, Xiayu Wei, Jin Wu, Xiaokun Li, Jin-San Zhang

**Affiliations:** ^1^School of Pharmaceutical Sciences, Wenzhou Medical University, Wenzhou, China; ^2^Qingdao University Medical College, Qingdao, China; ^3^The First Affiliated Hospital, Wenzhou Medical University, Wenzhou, China; ^4^Institute of Life Sciences, Wenzhou University, Wenzhou, China

**Keywords:** FGF10, ischemia-reperfusion, acute kidney injury, autophagy, inflammation, HMGB1

## Abstract

Ischemia-reperfusion (I/R) is a common cause of acute kidney injury (AKI), which is associated with high mortality and poor outcomes. Autophagy plays important roles in the homeostasis of renal tubular cells (RTCs) and is implicated in the pathogenesis of AKI, although its role in the process is complex and controversial. Fibroblast growth factor 10 (FGF10), a multifunctional FGF family member, was reported to exert protective effect against cerebral ischemia injury and myocardial damage. Whether FGF10 has similar beneficial effect, and if so whether autophagy is associated with the potential protective activity against AKI has not been investigated. Herein, we report that FGF10 treatment improved renal function and histological integrity in a rat model of renal I/R injury. We observed that FGF10 efficiently reduced I/R-induced elevation in blood urea nitrogen, serum creatinine as well as apoptosis induction of RTCs. Interestingly, autophagy activation following I/R was suppressed by FGF10 treatment based on the immunohistochemistry staining and immunoblot analyses of LC3, Beclin-1 and SQSTM1/p62. Moreover, combined treatment of FGF10 with Rapamycin partially reversed the renoprotective effect of FGF10 suggesting the involvement of mTOR pathway in the process. Interestingly, FGF10 also inhibited the release of HMGB1 from the nucleus to the extracellular domain and regulated the expression of inflammatory cytokines such as TNF-α, IL-1β and IL-6. Together, these results indicate that FGF10 could alleviate kidney I/R injury by suppressing excessive autophagy and inhibiting inflammatory response and may therefore have the potential to be used for the prevention and perhaps treatment of I/R-associated AKI.

## Introduction

Acute kidney injury is a global health concern. AKI is mainly caused by renal I/R injury, sepsis, and nephrotoxicant (such as cisplatin, cyclosporine and aristolochic acid) (Paller et al., 1984; Thadhani et al., 1996; Zuk and Bonventre, 2016). The primary characteristic of AKI is the rapid decline in kidney function as measured by detection of GFR (Bonventre and Yang, 2011; Havasi and Borkan, 2011). Despite advances in therapeutic strategies and nursing measures, including dialysis and kidney transplantation, the mortality of patients after AKI remains very high (Ueda et al., 2000; Chertow et al., 2005). In the past decades, AKI has been extensively studied both in clinic and experimental animal settings. The disease mechanisms underlying the etiology and pathogenesis of AKI are complex and include mitochondrial dysfunction, ROS, ER stress, autophagy, inflammation, apoptosis and necrosis (Basile et al., 2012; Tan et al., 2013, 2017; He et al., 2014; Kaushal and Shah, 2016; Xu et al., 2016). To date, there are no satisfying strategies or drugs for the therapy of patients with AKI.

A number of recent studies have demonstrated the crucial role of autophagy in animal models of AKI induced by I/R injury and nephrotoxic agents (Mizushima and Komatsu, 2011; Basile et al., 2012; Jiang et al., 2012; He et al., 2014; Guan et al., 2015; De Rechter et al., 2016; Lenoir et al., 2016). Autophagy is a highly conserved eukaryotic cellular recycling process by which cytoplasmic components are engulfed and degraded in the lysosome (Mizushima and Komatsu, 2011). Generally, autophagy is thought to be highly inducible under stress conditions such as ischemia, hypoxia, nutrient deprivation, genotoxic stress, infection, UPR, and other insults, all of which participate in the pathogenesis of AKI (Mizushima and Komatsu, 2011; Basile et al., 2012; He et al., 2014; De Rechter et al., 2016; Kaushal and Shah, 2016; Zuk and Bonventre, 2016). Whether autophagy is protective or damaging in AKI remains controversial. Renoprotective effects of autophagy in AKI have been reported in several studies (Pallet et al., 2008; Jiang et al., 2012). However, excessive activation of autophagy results in widespread cell death predominantly in RTCs due to extensive degradation of essential materials and organelles (Chien et al., 2007; Suzuki et al., 2008; Inoue et al., 2010). Therefore, activation of autophagy has dual roles in regulating cell survival or cell death in AKI.

Inflammatory response is another important component in the initiation and exacerbation of AKI. Although inflammation is an essential element of the body’s defense system, excessive activation of inflammatory cells and cytokine secretion impose severe damage to renal parenchyma cells (Jang and Rabb, 2009; Shibutani et al., 2015). High-mobility group box 1 is a member of the high-mobility group (HMG) protein family and one of the highly conserved and abundantly expressed proteins in almost all types of eukaryotic cells (Lotze and Tracey, 2005; Kang et al., 2014). Recently, the pathophysiological role of HMGB1 in human diseases has been extensively studied. In healthy circumstances, HMGB1 is localized in the nuclei of cells and participates in multiple cellular processes including DNA repair, transcription, and cell differentiation. However, HMGB1 can be released into the extracellular space and function as a signaling molecule in various biological processes such as inflammatory response (Tang et al., 2010a; Xu et al., 2014; Ouyang et al., 2016). Circulating HMBG1 is capable of engaging with toll-like receptors (TLRs), particularly TLR2 and TLR4, to activate the expression of multiple pro-inflammatory cytokines such as TNF-α, IL-1β and IL-6. Studies demonstrate that HMGB1 plays an important role in the interaction of autophagy and apoptosis/necrosis in various disorders including AKI (Nikoletopoulou et al., 2013; Kim et al., 2014; Chen et al., 2016).

Fibroblast growth factor 10, also known as Keratinocyte growth factor 2, is a typical paracrine FGF family member and signals through interactions with its high affinity receptor FGFR2-IIIb splicing isoform. FGF10 is a multifunctional growth factor playing crucial roles in the development of many organs and tissues including the kidney (Beenken and Mohammadi, 2009; Itoh, 2015). Deletion of either *Fgf10* or its receptor *Fgfr2-IIIb* in mice led to kidney dysgenesis characterized by fewer collecting ducts and nephrons (Bates, 2007). Overexpression of a dominant negative receptor isoform in transgenic mice has revealed more striking defects including renal aplasia or severe dysplasia (Bates, 2007). Recent studies have reported the protective effect of FGF10 on spinal cord injury, cerebral ischemia injury and acute lung injury via inhibiting inflammation, activating PI3K/AKT signaling pathway or mobilization of stem cells (Li et al., 2016; Tong et al., 2016; Chen et al., 2017). Currently, there are no published reports regarding whether exogenous FGF10 can promote the recovery of AKI. In the present work, we tested the hypothesis that FGF10 administration might protect renal cells exposed to I/R injury through regulating autophagy and inflammation.

## Materials and Methods

### Reagents and Antibodies

Recombinant human FGF10 was acquired from Zhejiang Grost Biotechnology (Wenzhou). Antibodies against mTOR, LC3, SQSTM1, Beclin-1 and GAPDH were purchased from Santa Cruz Biotechnology (Santa Cruz, CA, United States). Antibodies against cleaved Caspase-3, HMGB1, phospho-FGFR, TNF-α and Caspase-9 were bought from Cell Signaling Technology (Beverly, MA, United States). TGF-β antibody was purchased from Abcam (Cambridge, MA, United States). The autophagy inhibitor chloroquine, autophagy activator rapamycin and 4′, 6-diamidino-2-phenylindole (DAPI) were purchased from Sigma-Aldrich (St Louis, MO, United States) and Invitrogen (Carlsbad, CA, United States), respectively.

### Animals

Adult male Sprague Dawley (SD) rats (8–12 weeks old) were supplied by Shanghai SLAC Laboratory Animal Co., Ltd., and housed in SPF facility of Wenzhou Medical University. The protocols for all animal experiments were approved by the institutional Animal Care and Use committee. Rats were anesthetized with intra-peritoneal injection of 4% pentobarbital sodium (50 mg/kg, Merck, Germany) and underwent right nephrectomy followed by ischemia for 60 min with renal artery clamping. SD rats were randomly divided into four groups: (I) Sham group: the left kidney was exposed with an unrestricted renal artery; (II) I/R group: the left kidneys were subjected to 60 min of ischemia by renal artery clamping followed by reperfusion (Kalogeris et al., 2012; Tan et al., 2017); (III) I/R-FGF10 group: a single dose of FGF10 (0.5 mg/kg) was injected into the abdominal cavity 30 min before the 60 min exposure to ischemia; (IV) RAPA group: a single dose of rapamycin (10 mg/kg, intramuscular injection, i.m) was injected followed by the injection of FGF10 same with I/R-FGF10 group, and then the left kidneys were subjected to 60 min of ischemia. For combined treatment with chloroquine (I/R-CL group): a single dose of chloroquine (60 mg/kg) was injected into the abdominal cavity 30 min before the 60 min exposure to ischemia. Animals were sacrificed at indicated time points after reperfusion upon surgical operation and kidneys were harvested for further experiment.

### Renal Function and Histopathology

Serum creatinine and BUN were used to assess changes of renal function after AKI. The levels of SCr and BUN were determined by the Creatine and the Urease colorimetry methods, respectively, which were performed at the Medical Laboratory Center of the First Affiliated Hospital, Wenzhou Medical University. For renal histology analysis, Kidneys were dissected and fixed with 10% formaldehyde for 48 h, then embedded in paraffin. To access the severity of renal injury after AKI, sections (5 μm) were stained with H&E to observe the changes of the renal morphology.

#### Immunohistochemistry and Immunofluorescent Staining

The slides were incubated with antibodies against cleaved-Capase-3, p-FGFR, SQSTM1 and TNF-α at 4°C overnight and stained with Diaminobenzidine (DAB) and counterstained with hematoxylin. The slides were then subjected to gradient ethanol dehydration, dimethyl benzene transparent, and mounted with Neutral resin cover slides. Images were captured using a Nikon ECLPSE 80i. For immunofluorescent staining, 5 μm sections were incubated at 4°C overnight with primary antibody against LC3, Beclin-1 and HMGB1, respectively. The slides were then incubated with donkey anti-rabbit secondary antibodies (Abcam, MA, United States) or donkey anti-mouse IgG-PE secondary antibodies (Santa Cruz, CA, United States) for 1 h at room temperature. The images were captured using a laser confocal microscope (Nikon, Ti-E&A1 plus).

### Apoptosis Assay

To measure the apoptosis rates after I/R injury, DNA fragmentation *in vivo* was detected using a one-step TUNEL Apoptosis Assay KIT (Roche, Mannheim, Germany) as previously described (Tan et al., 2017). The images were captured with a Nikon ECLPSE Ti microscope (Nikon, Japan).

### Western Blot Analysis

Tissue protein samples were prepared with protein extraction reagents from renal tissues. Protein concentrations were measured with a Pierce BCA Protein Assay Kit (Thermo Fisher Scientific). Samples with equal amount of proteins were separated with SDS-PAGE and then transferred onto a PVDF membrane for Western blot analysis with specified antibodies. The ChemiDic TM XRS+ imaging system (Bio-Rad Laboratories, Hercules, United States) was used to analyze the signals and the band densities were quantified with Multi Gauge software of science Lab 2010 (FUJIFILM Corporation, Tokyo, Japan).

### Real-Time Quantitative RT-PCR

Total RNA from kidney tissues was extracted using RNeasy column (QIAGEN), and reverse transcription was performed using Prime Script TM RT reagent Kit (TaKaRa) according to the manufacturer’s instructions. Real-time RT-PCR was performed using the SYBR Green gene expression assays (TaKaRa) to access mRNA expression. The target values were normalized to GAPDH (Tan et al., 2017). The PCR primers used for mRNA expression analysis of *Tlr2, Tlr4, Il-Iβ, Il-6*, and *Gapdh* are summarized in Table 1.

**Table 1:** Primer sequences used to amplify rat cDNAs.

Gene	GenBank	Primer sequences
GAPDH	NM_012675	5′- GACATGCCGCCTGGAGAAAC-3′
		5′-AGCCCAGGATGCCCTTTAGT-3′
IL-1β	NM_031512	5′-TGCAGGCTTCGAGATGAAC-3′
		5′-GGGATTTTGTCGTTGCTTGTC-3′
IL-6	NM_012589	5′-AAGCCAGAGTCATTCAGAGC-3′
		5′-GTCCTTAGCCACTCCTTCTG-3′
TLR2	NM_198769	5′-ATGAACACTAAGACATACCTGGAG-3′
		5′-CAAGACAGAAACAGGGTGGAG-3′
TLR4	NM_019178	5′-CATGACATCCCTTATTCAACCAAG-3′
		5′-GCCATGCCTTGTCTTCAATTG-3′
TNFα	NM_012675	5′-CTTCTCATTCCTGCTCGTGG-3′
		5′-TGATCTGAGTGTGAGGGTCTG-3′


*GAPDH: Glyceraldehyde 3-phosphate dehydrogenase; IL-1β: interleukin-1β; IL-6: interleukin-6; TLR2: Toll-like receptor-2; TLR4: Toll-like receptor-4; TNFα: tumor necrosis factor-α.*

### Statistical Analysis

Data is expressed as the mean ± SEM of independent experiments (*n* ≥ 5). Statistical significance was determined using Student’s *t*-test when there were two experimental groups. When more than two groups were compared, statistical evaluation of the data was performed using one-way analysis of variance (ANOVA). ^∗^*P* < 0.05, ^∗∗^*P* < 0.01, ^∗∗∗^*P* < 0.001, *P-*values < 0.05 was regarded as statistically significant.

## Results

### FGF10 Ameliorates I/R-Induced Renal Dysfunction and Histological Damage

**FIGURE 1:**
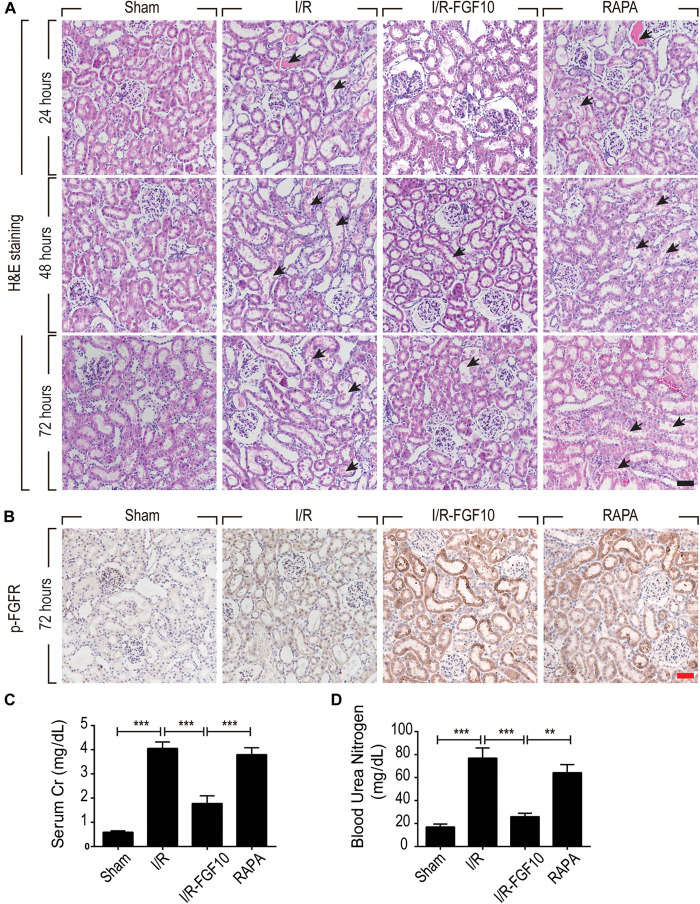
FGF10 protects against renal histological and function damage after I/R injury. **(A)** Histological changes of kidneys detected by H&E staining at 24, 48, and 72 h, respectively, after reperfusion. Animals were randomly assigned into 4 groups: namely, Sham group, I/R group, I/R-FGF10 group and RAPA group. The details of operations and treatment animals received were described in the materials and methods. Arrows show intraluminal necrotic cells. Scale bars = 50 μm. **(B)** IHC staining for p-FGFR in renal tissue sections of indicated groups. Scale bars = 50 μm. **(C,D)** Determination of SCr and BUN levels in the above grouped rats at 2 days after reperfusion (mean ± SEM; *n* = 5). ^∗∗^*P* < 0.01, ^∗∗∗^*P* < 0.001.

We employed an I/R injury rat model to investigate the potential effect of FGF10 on AKI at 24, 48, and 72 h, respectively. Renal histological changes were assessed by H&E staining, no apparent damage was observed in the kidney of sham group, whereas the rats in I/R group and RAPA group showed swelling of RTCs, intraluminal necrotic cellular debris, interstitial congestion and luminal narrowing characteristic of I/R-induced tubular epithelial injury at each time point after reperfusion (Figure 1A). Significantly, Pre-administration of FGF10 markedly attenuated the degree of renal damages and largely preserved the normal tissue architecture and integrity. Renal function was assessed by measuring SCr and BUN at 48 h after reperfusion. As expected, the levels of SCr and BUN were both increased significantly in I/R rats compared to Sham group (Figures 1C,D). Notably, the levels of SCr and BUN in I/R-FGF10 group were significantly lower compared to that of I/R group (*P* < 0.001), whereas Rapamycin largely abolished the protective effect of FGF10 against I/R injury. To investigate the association between FGF10/FGFR signaling pathway and I/R injury, we detected the activation of FGFR by immunohistochemistry (IHC) staining with p-FGFR antibody. As shown in Figure 1B, few p-FGFR positive cells were detected in kidneys of sham group, whereas the number of p-FGFR positive cells was increased in I/R group at 48 h after reperfusion. However, both the number of p-FGFR positive renal tubular cells (RTCs) and the staining intensity were noticeably increased in kidneys of I/R-FGF10 group or RAPA group compared to I/R group.

### FGF10 Reduced Apoptosis of RTCs via Regulation of Pro-apoptotic Proteins

**FIGURE 2:**
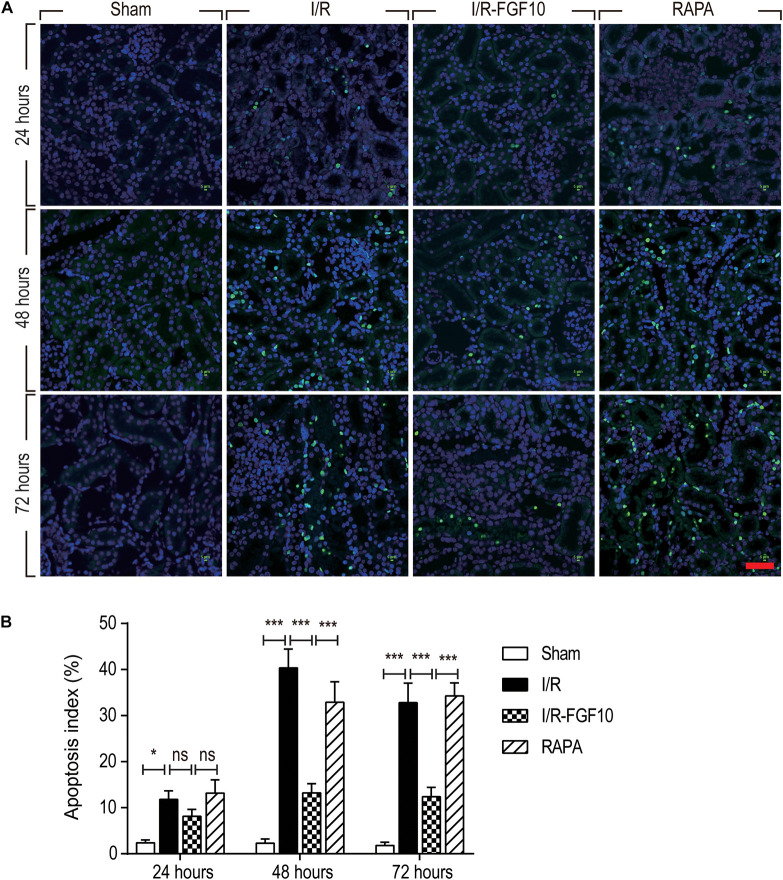
FGF10 protects against I/R induced apoptosis in RTCs. **(A)** Representative sections of nuclear DNA fragmentation staining were performed using TUNEL in different groups at 24, 48, and 72 h, respectively, after reperfusion. Scale bars = 50 μM. **(B)** Quantitative analysis of the number of TUNEL-positive RTCs. Data are presented as the mean ± SD (*n* = 5). ^∗^*P* < 0.05, ^∗∗∗^*P* < 0.001. The percentage of positive cells was analyzed with 5 individual magnification × 400 fields per group.

TUNEL staining was carried out to assess the apoptosis in RTCs. As shown in Figure 2A, compared to the sham group, the number of TUNEL-positive cells in I/R rats was dramatically increased (*P* < 0.001). Significantly, the proportion of TUNEL-positive cells was much lower in I/R-FGF10 group (*P* < 0.001). However, this apparent effect of FGF10 against I/R-induced apoptosis was mostly antagonized by rapamycin treatment (Figure 2A). The number of TUNEL-positive cells was strikingly increased in RAPA group compared to the IR-FGF10 group. Quantification analysis of TUNEL staining revealed that the average percentage of apoptotic cells were 2.40% (24 h), 2.64% (48 h) and 1.92% (72 h) in sham group; 11.8% (24 h), 40.34% (48 h), 32.8% (72 h) in I/R group; 8.14% (24 h), 13.22% (48 h), 12.38% (72 h) in I/R-FGF10 group and 13.2% (24 h), 32.9% (48 h), 34.28% (72 h) in RAPA group, respectively (Figure 2B). The results indicated that FGF10 treatment protected RTCs from I/R-induced apoptosis based on TUNEL staining. However, the protective role of FGF10 against apoptosis was diminished by rapamycin.

**FIGURE 3:**
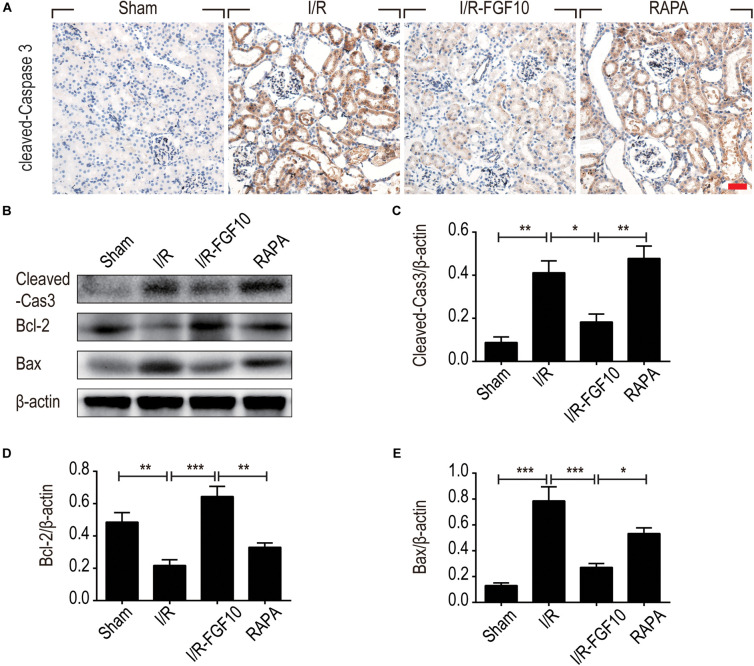
FGF10 reduces the expression of pro-apoptotic proteins IHC staining and Western blot analyses were performed at 2 days after reperfusion. **(A)** IHC staining for cleaved caspase-3 in kidneys of indicated groups. Scale bars = 50 μm. **(B)** The expression of cleaved Caspase-3, Bcl-2 and Bax were detected by Western blot with β-actin as loading control. **(C–E)** The optical density analysis of cleaved Caspase-3, Bcl-2 and Bax (mean ± SEM; *n* = 5). ^∗^*P* < 0.05, ^∗∗^*P* < 0.01, ^∗∗∗^*P* < 0.001.

To understand the protective mechanism of FGF10 against I/R-induced RTC apoptosis, we examined the expression of pro-apoptotic proteins involved in regulation of cell apoptosis (BCL-2, BAX) and cleaved-Caspase-3 by IHC staining (Figure 3A) and western blot (Figures 3B–E), respectively. The expression of BAX and cleaved-Caspase-3 were significantly increased upon I/R injury, whereas BCL2 expression was decreased. Significantly, FGF10 treatment inhibited the pro-apoptotic expression/activation of Bax/BCL2 and cleaved-Caspase-3, respectively. Consistent with the results of apoptosis, the effect of FGF10 was largely inhibited by co-treatment with rapamycin. Together, the results suggest that FGF10 protects RTCs from I/R-induced apoptosis via regulation of pro-apoptotic proteins. However, rapamycin inhibited the role of FGF10 and thus the expression of pro-apoptotic proteins was increased.

### The Protective Effect of FGF10 Is Related to the Regulation of Autophagy via mTOR Pathway

Autophagy is known to play a crucial role in the etiology of AKI caused by renal I/R injury. The fact that rapamycin, a well-established allosteric mTOR kinase inhibitor and agonist of autophagy, mostly reduced the protective effect of FGF10 against I/R-induced renal damage apoptosis of RTCs prompted us to further examine the involvement of autophagy in mediating protective effect of FGF10.

**FIGURE 4:**
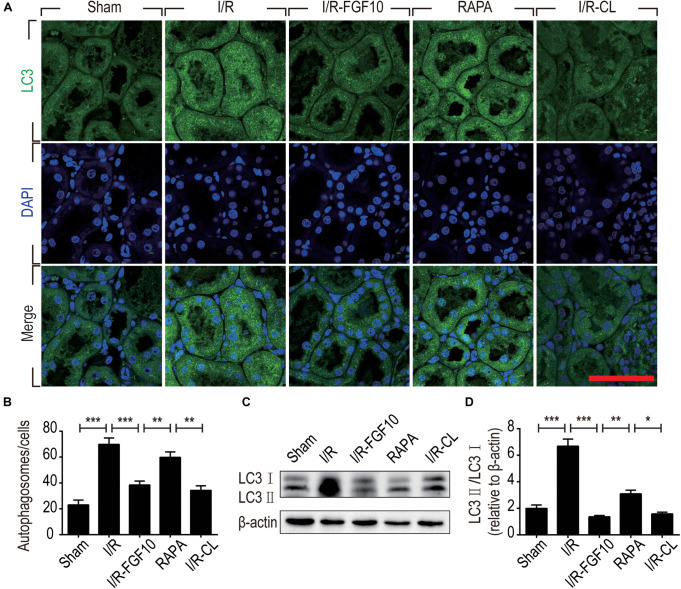
FGF10 reduces the formation of autophagosome and the expression of LC3II. **(A)** Immunofluorescence staining of LC3 (green) was performed at 48 h after reperfusion. Nuclei were labeled with DAPI (blue). Scale bars = 50 μm. **(B)** Statistic analysis of the number of autophagosomes in RTCs with 5 randomly selected images in each group. **(C)** The protein expression of LC3II/LC3I in renal tissue was determined by Western blot and the optical densities were quantified **(D)**. Data are presented as the mean ± SEM (*n* = 5). ^∗^*P* < 0.05, ^∗∗^*P* < 0.01, ^∗∗∗^*P* < 0.001.

**FIGURE 5:**
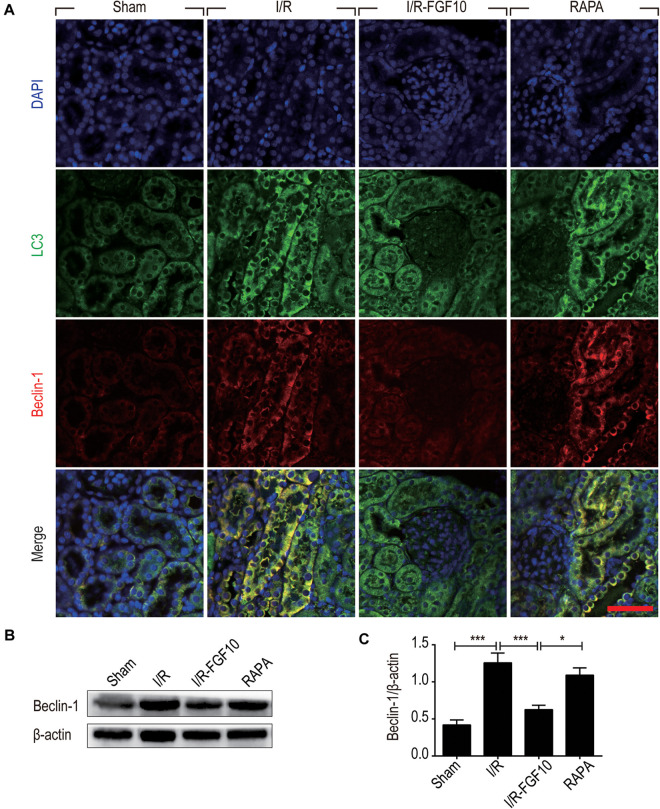
FGF10 reduces the expression of Beclin-1. **(A)** Immunofluorescence staining and confocal images for LC3 (Green) and Beclin-1 (red) at 2 days after reperfusion. Nuclei were labeled with DAPI (blue). Scale bars = 50 μm. **(B)** Representative western blotting result for Beclin-1 expression. **(C)** Optical density analysis of protein bands. Data are presented as the mean ± SEM (*n* = 5). ^∗^*P* < 0.05, ^∗∗∗^*P* < 0.001.

Detection of LC3I to LC3II conversion and expression of Beclin-1 and SQSTM1/p62 (SQSTM1 is used hereafter) remains the most reliable methods to gauge autophagic activity. We therefore examined the expression of LC3, Beclin-1 and SQSTM1 at tissue and protein levels by immunofluorescence staining and immunoblot, respectively. The confocal imaging in Figure 4A shows that the number of LC3 positive dots (autophagosomes) were dramatically increased in the I/R group compared to sham group, but greatly reduced by FGF10. Rapamycin treatment effectively abolished the effect of FGF10 in this setting. However, chloroquine, as a specific autophagy inhibitor, markedly reduced the number of autophagosomes in RTCs caused by I/R injury. The statistical analysis about the number of autophagosomes in each group was shown in Figure 4B. This result is confirmed with immunoblot analysis showing that I/R induced LC3II was partially prevented by FGF10 treatment (Figure 4C, and quantification result in Figure 4D). Co-detection of Beclin-1 and LC3 by immunofluorescence staining also revealed that increased expression of Beclin-1 in I/R tissues was largely prevented by FGF10, an effect also reversed by treatment with rapamycin (Figure 5A). Western blot detection and quantification analysis on Beclin-1 expression (shown in Figures 5B,C) revealed a similar trend of alteration to LC3II (Figures 4C,D), and was consistent with confocal image analysis (Figures 4A, 5A).

**FIGURE 6:**
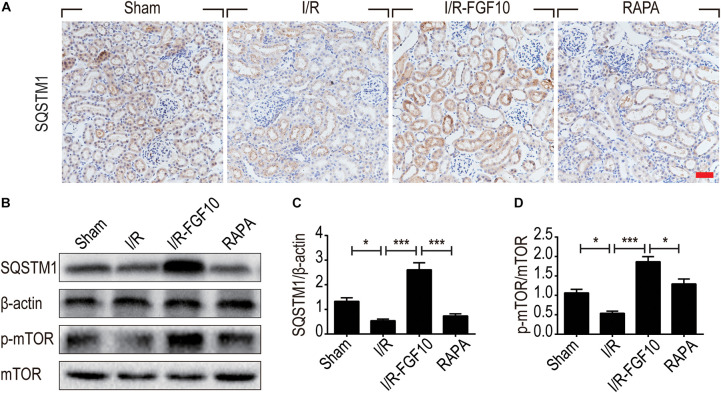
FGF10 increases the expression of SQSTM1 and p-mTOR in I/R rats. **(A)** IHC staining was performed at 2 days after reperfusion for SQSTM1 in kidney tissues from indicated animal groups. Scale bars = 50 μm. **(B)** The expression of SQSTM1, p-mTOR and mTOR were detected by western blotting (mean ± SEM; *n* = 5). β-actin was used as control. ^∗^*P* < 0.05, ^∗∗∗^*P* < 0.001. **(C,D)** Optical density analysis for SQSTM1 and p-mTOR, which were normalized to β-actin and mTOR, respectively.

Besides LC3II and Beclin-1, we also examined the expression of SQSTM1, a selective autophagic receptor and substrate. As shown in Figure 6A, SQSTM1 was expressed in the cytoplasm of RTCs, which was significantly decreased in I/R group. It was evident that FGF10 not only reversed I/R-induced decrease of SQSTM1, but further increased its expression above the one observed for the sham control (Figures 6B,C), this effect again was abolished by rapamycin. To determine whether the mTOR pathway is subjected to FGF10 regulation, we examined the phosphorylation of mTOR by immunoblot. As shown in Figure 6B, the changes in phosphorylation of mTOR highly resembled that of SQSTM1, which was decreased in I/R group, but became markedly increased in FGF10 treated group, an effect mostly inhibited by co-treatment with rapamycin (Figure 6D).

### FGF10 Inhibited the Release of HMGB1 in Response to Renal I/R Injury

**FIGURE 7:**
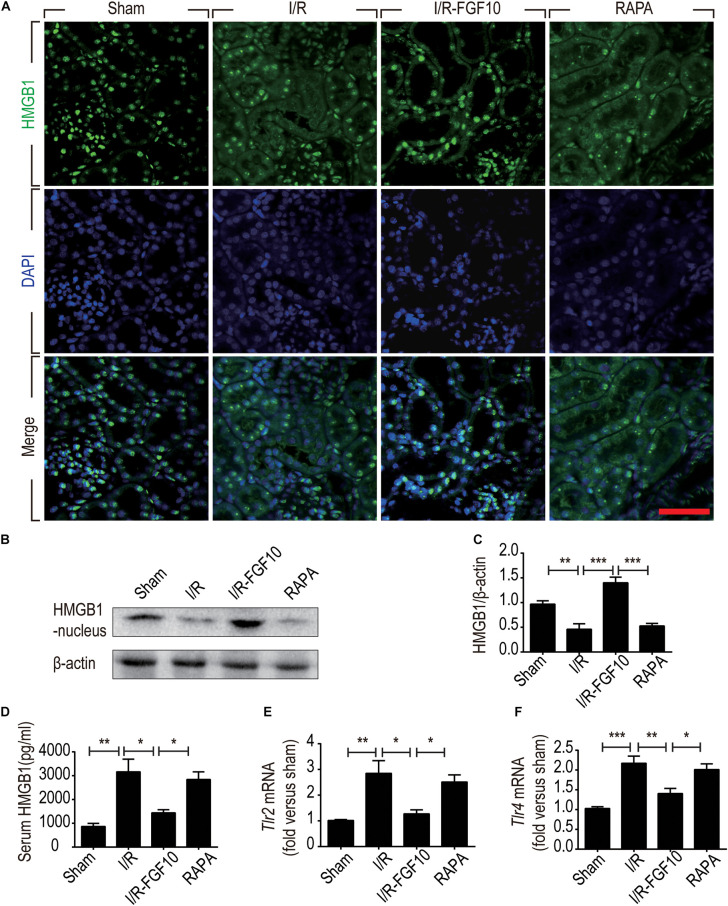
FGF10 inhibits the release of nuclear HMGB1 to the serum and regulates the TLR mRNA expression. **(A)** Immunofluorescence staining of HMGB1 at 2 days after reperfusion. Nuclei were labeled with DAPI (blue). Scale bars = 50 μm. **(B,C)** Protein expression of HMGB1 in the nuclear fraction of renal tissues by Western blot and optical density analysis with β-actin as loading control (mean ± SEM; *n* = 5). ^∗∗^*P* < 0.01, ^∗∗∗^*P* < 0.001. **(D)** Level of serum HMGB1 was determined by ELISA (mean ± SEM; *n* = 5). ^∗^*P* < 0.05, ^∗∗^*P* < 0.01. **(E,F)** Expression of Tlr2 and Tlr4 mRNA in the kidney were examined by RT-qPCR and normalized to Gapdh. ^∗^*P* < 0.05, ^∗∗^*P* < 0.01, ^∗∗∗^*P* < 0.001.

HMGB1 is a major DAMP protein, which can be activated by renal I/R and participates in inflammatory response (Wu et al., 2010). We therefore examined the expression and localization of HMGB1 by Immunofluorescence staining and confocal imaging analyses. As expected, HMGB1 was predominantly localized in the nuclei of RTCs in sham control. Following I/R injury, the level of HMGB1 appeared to be decreased in nuclei, but increased in the cytoplasmic domain. Strikingly, FGF10 almost completely prevented the decrease of nuclear HMGB1 and concomitant increase in the cytoplasm, an effect abolished by rapamycin treatment (Figure 7A). To confirm the nucleus to cytoplasm shuttling and extracellular release of HMGB1, we further examined the levels of nuclear as well as serum HMGB1 by western blot and ELISA, respectively (Figures 7B–D). The expression of HMGB1 in the nuclear fraction was significantly decreased, whereas the serum HMGB1 was significantly increased in I/R group compared with sham group. FGF10 treatment completely prevented the I/R-induced decrease of nuclear HMGB1 (Figures 7B,C), and largely abolished the increase in serum HMGB1 (Figure 7D). Extracellular HMGB1 is known to signal through TLRs, particularly TLR2 and TLR4, to activate pro-inflammatory response. Indeed, we found that the level of *Tlr2* mRNA expression was increased nearly threefold against sham-operated rats (Figure 7E). Importantly, FGF10 treatment mostly obliterated I/R-induced *Tlr2* expression, an effect partially reversed by rapamycin treatment. The effect of FGF10 on the mRNA expression of *Tlr4* was similar to that of *Tlr2* (Figure 7F). These results provide evidence that FGF10 could inhibit the release of HMGB1 from the nucleus to the extracellular matrix thereby preventing the HMGB1-mediated inflammatory response via the TLR2/TLR4 signaling pathway.

### FGF10 Inhibited the Expression of Inflammatory Cytokines After I/R Injury

**FIGURE 8:**
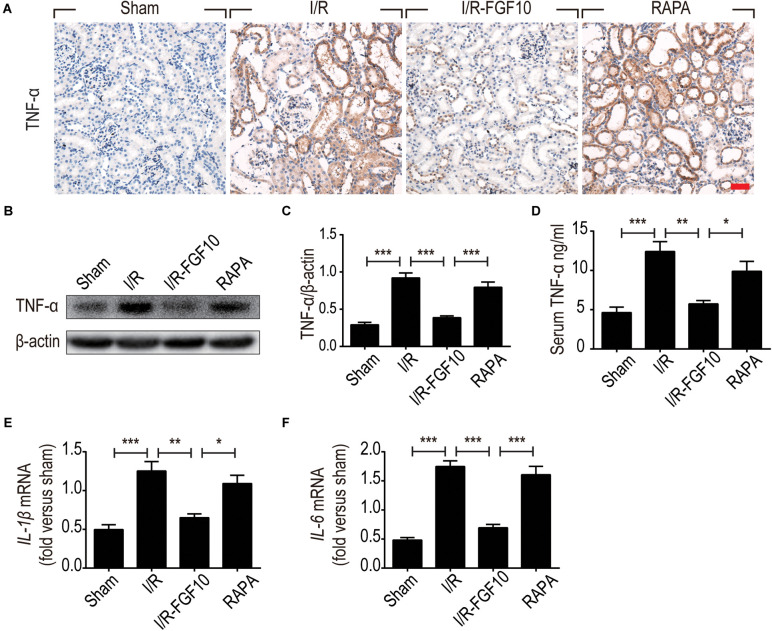
FGF10 regulates the expression of inflammatory cytokines. **(A)** IHC staining for TNF-α in kidney tissues from indicated groups. Scale bars = 50 μm. **(B,C)** The expression of TNF-α was detected by western blot using TNF-α specific antibody. Optical density analyses (mean ± SEM; *n* = 5) with β-actin as control. **(D)** Levels of serum TNF-α were determined by ELISA (mean ± SEM; *n* = 5). ^∗^*P* < 0.05, ^∗∗^*P* < 0.01, ^∗∗∗^*P* < 0.001. **(E,F)** The mRNA expression of Il-1β and Il-6 in the kidney was examined by RT-qPCR and normalized to Gapdh. ^∗^*P* < 0.05, ^∗∗^*P* < 0.01, ^∗∗∗^*P* < 0.001.

The ability of FGF10 to prevent I/R induced HMGB1 nuclear to cytoplasmic shuttling and releases, as well as TLR2 induction in response to I/R injury suggests that FGF10 may inhibit the expression of pro-inflammatory cytokines such as TNF-α. We therefore examined the expression of TNF-α in kidneys by IHC staining (Figure 8A) and western blot (Figures 8B,C). The serum TNF-α was also examined by ELISA (Figure 8D). I/R-induced TNF-α expression was mostly prevented by FGF10, but such effect, was largely obliterated by rapamycin treatment. We next performed RT-qPCR to determine the mRNA expression of two other inflammatory cytokines *Il-1*β and *Il-6* in renal tissues. These results also demonstrated that I/R-induced expression of these cytokines could be effectively inhibited by FGF10, but not in the presence of rapamycin (Figures 8E,F).

## Discussion

FGF10, a multifunctional growth factor, is crucial in transmitting mesenchymal to epithelial signaling in organ development and regenerative medicine (Itoh, 2016). The role of FGF10 in cerebral ischemia injury, pulmonary fibrosis and wound healing, has been extensively researched (Li et al., 2016; Tong et al., 2016; Chao et al., 2017; Chen et al., 2017; El Agha et al., 2017). As a typical paracrine growth factor, FGF10 and its predominant receptor FGFR2-IIIb plays crucial roles in the development of kidney. However, the potential effect of FGF10 on AKI has not been reported so far. We herein used a well-established renal I/R model to investigate the potential protection effect of FGF10 against I/R injury. We confirmed that I/R rats were associated with increased SCr and BUN indicating a decline in the GFR. The current work provided experimental evidence that FGF10 administration effectively alleviated I/R-induced functional impairment as well as histological damage of the kidney. Mechanistically, besides curbing apoptosis induction in RTCs, administration of FGF10 effectively alleviated the excessive autophagy, a common phenotype in RTCs exposed to I/R injury. HMGB1 is a damage-associated molecule that is rapidly released from nucleus to extracellular matrix and acts as a crucial molecule in the mediation of apoptosis and inflammation. We here demonstrated that FGF10 can inhibit the translocation of HMGB1 and thus attenuates RTC apoptosis upon I/R injury. Therefore, FGF10 treatment appears to protect kidneys from AKI via the regulation of autophagy and HMGB1 mediated inflammatory signaling pathways.

Extensive research has demonstrated that death of renal parenchyma cells, including apoptosis and necrosis, is the major mechanism underlying the pathogenesis of the AKI, as well as inflammation (Oberbauer et al., 2001). The RTCs detached from basement membrane, along with other cellular debris, enter and obstruct the tubular lumen, thereby decrease GFR. Upon examining multiple parameters of cell death including TUNEL assays which detects both apoptosis and necrosis, as well as crucial mitochondrial regulators, we conclude that FGF10 treatment ameliorates the pro-apoptotic alteration of Bax/Bcl-2 as well Caspase-3, therefore RTC apoptosis following I/R injury. The results are in line with our previous studies showing that FGF2, another FGF family member, protects against renal I/R injury through attenuating mitochondrial damage (Tan et al., 2017). Two recent studies suggested that neuron and microglia or macrophage-derived FGF10 participates in activation of PI3K/AKT/mTOR, which contributes to either ameliorate cerebral ischemia injury or improve functional recovery after spinal cord injury (Li et al., 2016; Chen et al., 2017). Further studies will be required to delineate the molecular mechanisms underlying FGF10 mediated protection against renal I/R injury.

A number of reports have established the involvement of autophagy in I/R-induced AKI in various animal models. In a myocardial I/R model, FGF2 is shown to improve heart function recovery and survival of cardiomyocytes through inhibition of excessive autophagy and increased ubiquitinated protein clearance via the activation of PI3K/AKT/mTOR signaling (Wang et al., 2015). Under normal physiological conditions, basal autophagy is required to maintain homeostasis in both visceral epithelial cells (podocytes) and RTCs. So far, both the beneficial and detrimental effects of autophagy have been reported after renal I/R injury in animal experiments (Decuypere et al., 2015; Kaushal and Shah, 2016; Lenoir et al., 2016). Autophagy has been reported to have a protective role in cell survival by degrading misfolded/unfolded proteins, damaged organelles and generate necessary nutrient substance during AKI in some reports (Kimura et al., 2011; Guan et al., 2015; Xie et al., 2018), whereas in others, it also causes apoptosis through excessive degradation of essential proteins and digestion of organelles (Shintani and Klionsky, 2004; Thorburn, 2008). Therefore, the role of autophagy in the pathogenesis and resolution of AKI injuries remains controversial, and is likely affected by the cellular context and also the extent of injury (Huber et al., 2012).

Given that rapamycin, an mTOR inhibitor and agonist of autophagy, impaired the protective effect of FGF10 on renal function, we therefore further examined several well-established autophagy parameters by immunoblot, immunofluorescence staining and associated autophagic phenotypes. LC3 is a crucial cytoplasmic protein required for the formation and elongation of autophagosome. LC3 positive punctate formation and the conversation of LC3I to LC3II are often used to examine the induction of autophagy. Our immunofluorescent analysis of LC3 indicated that FGF10 treatment could prevent I/R-induced conversion of LC3I to LC3II and inhibited the formation of autophagic vacuoles and autophagosome. More strikingly, the effect of FGF10 on I/R-induced autophagy was nearly completely antagonized by rapamycin therefore establishing a role of autophagy in the protective effect of FGF10 again I/R injury. Consistent results were observed with the expression of Beclin-1, a marker of autophagosome as well as SQSTM1, an ubiquitously expressed protein which directly interacts with LC3 and subsequently degraded in autophagosome (Johansen and Lamark, 2011; Weidberg et al., 2011). The decreased expression of SQSTM1 upon I/R injury was partially restored by FGF10 treatment. The data collectively suggest that FGF10 treatment could reduce autophagosome formation and inhibit excessive autophagy in RTCs after I/R injury via mTOR pathway.

Although both apoptosis and autophagy are rapidly induced in RTCs during AKI, but the role of autophagy in AKI is not as clear as apoptosis, and the interaction between apoptosis and autophagy in response to stimuli is complex and poorly defined. It is generally accepted that moderate autophagy may enhance the cellular ability to cope with stress response and thus promotes cell survival. Several studies have reported the renoprotective effect of autophagy in AKI caused by 25–40 min of renal ischemia-reperfusion (Kimura et al., 2011; Jiang et al., 2012; Xie et al., 2018). Once the autophagy is exacerbated due to severe injury, the program of apoptosis would be activated and eliminate the irreversibly damaged cells. Our results clearly indicate that FGF10 treatment could alleviate the excessive autophagy induced by 60 min of I/R exposure and thus protects RTCs from apoptosis. Therefore the extent of renal injury may render autophagy to either alleviate or augment the I/R injury. However, no study has shown a definite demarcation point to distinguish the dual roles of autophagy on damaged cells. The regulatory mechanism between autophagy and apoptosis in response to I/R injury should be a focus of future studies.

The innate immune response is another integral pathological mechanism with AKI and the subsequent CKD. Emerging evidence suggests that the relationship between autophagy and inflammation is far more complicated than previously appreciated (Leventhal et al., 2014). Both autophagy and immune response play crucial roles in the pathogenesis of AKI. Immune responses can affect the activation and perpetuation of autophagy in RTCs after reperfusion. Autophagy is identified a modulator that can both regulate and be regulated by immune responses in many diseases (Kaushal and Shah, 2016; Kimura et al., 2017). Further research is needed to clarify the precise effects of autophagy on inflammation.

Many studies reported the multiple roles of HMGB1 in the pathogenesis of various diseases. However, the crosstalk between HMGB1 and apoptosis is complicated and requires further elucidation. HMGB1 shows dual roles in the regulation of apoptosis. Intracellular HMGB1 is generally an anti-apoptosis molecule, whereas overexpression of extracellular HMGB1 promotes apoptosis (Kang et al., 2014). The two-way interaction between HMGB1 and autophagy has been wildly studied. Autophagy participates in various physiological and pathological processes including the release and degradation of HMGB1 (Thorburn et al., 2009; Dupont et al., 2011). Autophagy is regulated by HMGB1 which involves many molecules such as heat shock protein β-1 (HSPB1), Bcl-2 and Beclin-1 (Tang et al., 2010b; Zhao et al., 2011). Studies with HMGB1 knockout mice suggest that loss of HMGB1 leads to autophagy deficiency, whereas increased extracellular HMGB1 promotes autophagy through binding to Receptor for advanced glycation end products (RAGE), a negative regulator of apoptosis (Tang et al., 2010a; Yanai et al., 2013). HMGB1 participates in the formation of renal fibrosis in the development of CKD through binding to TLR2 and RAGE. Therefore, future studies are warranted to explore the effect of FGF10 on CKD.

In summary, the present study demonstrates for the first time that exogenously administered recombinant FGF10 protects against I/R-induced functional and tissue damage to the kidney. The potent protective effect is attributed to its ability to attenuate several I/R-induced pro-apoptotic alteration of BCL2/BAX expression and Caspase-3 activation, therefor apoptotic cell death of renal parenchyma cells. The present work also indicates that protective effect of FGF10 against I/R injury is related to its down-regulation of excessive autophagy as well as release of HMGB1, Which in turn regulates pro-inflammatory immune response via TLR2/TLR4 signaling pathway. Apoptosis and autophagy are both rapidly activated upon renal I/R injury, which may interact with each other to govern the pathological and recovery processes of AKI. Our study suggests that FGF10 may provide a potential therapeutic option for treating AKI.

## Author Contributions

XT and J-SZ conceived and designed the experiments. XT performed the animal operations and acquired the confocal images. HZ performed apoptosis assay, immunoblot, immunohistochemistry, and immunofluorescent staining. QT, LG, TJ, LX, XW, JW, and RY assisted in animal housing effort and acquisition of some of the experimental data related to RT-PCR, and ELISA. XT and HZ analyzed the data and prepared the figures. XT and J-SZ wrote and revised the manuscript. J-SZ and XL supervised and funded the study.

## Conflict of Interest Statement

The authors declare that the research was conducted in the absence of any commercial or financial relationships that could be construed as a potential conflict of interest.

## Abbreviations

AKI: acute kidney injury
ANOVA: analysis of variance
BUN: blood urea nitrogen
CKD: chronic kidney disease
DAMP: damage-associated molecular pattern
ELISA: enzyme-linked immunosorbent assay
ER: endoplasmic reticulum
FGF10: fibroblast growth factor 10
GFR: glomerular filtration rate
H&E: hematoxylin and eosin
HMGB1: high-mobility group box 1
I/R: ischemia-reperfusion
mTOR: mammalian target of rapamycin
PVDF: polyvinylidene fluoride
ROS: reactive oxygen species
RTCs: renal tubular epithelial cells
SCr: serum creatinine
SDS-PAGE: sodium dodecyl sulfate–polyacrylamide gel electrophoresis
TLRs: Toll-like receptors
TUNEL: terminal deoxynucleotidyl transferase dUTP nick end labeling
UPR: unfolded protein response
