# Corrigendum: FGF10 Protects Against Renal Ischemia/Reperfusion Injury by Regulating Autophagy and Inflammatory Signaling

**DOI:** 10.3389/fgene.2021.731406

**Published:** 2021-11-12

**Authors:** Xiaohua Tan, Hongmei Zhu, Qianyu Tao, Lisha Guo, Tianfang Jiang, Le Xu, Ruo Yang, Xiayu Wei, Jin Wu, Xiaokun Li, Jin-San Zhang

**Affiliations:** ^1^ School of Pharmaceutical Sciences, Wenzhou Medical University, Wenzhou, China; ^2^ Qingdao University Medical College, Qingdao, China; ^3^ The First Affiliated Hospital, Wenzhou Medical University, Wenzhou, China; ^4^ Institute of Life Sciences, Wenzhou University, Wenzhou, China

**Keywords:** FGF10, ischemia-reperfusion (I/R), acute kidney injury, autophagy, inflammation, HMGB1

In the original article, there were mistakes in Figure 2A, Figure 6A, and Figure 7A as published. The immunofluorescence and immunohistochemistry images in the Sham group (Figure 2A) and RAPA groups in Figure 6A and Figure 7A, respectively, were erroneously used. The corrected Figures appear below.

**FIGURE 2:**
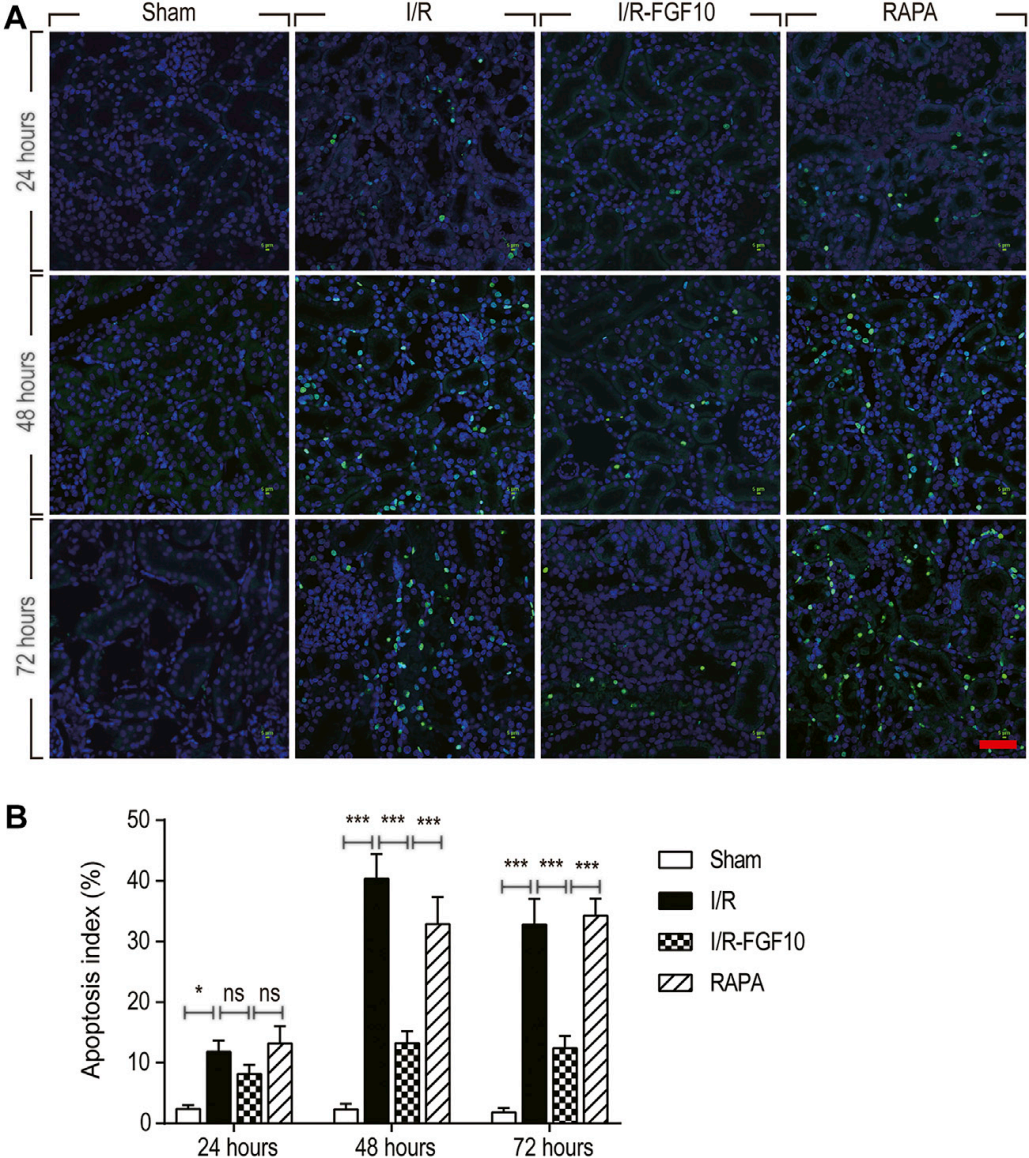
FGF10 protects against I/R induced apoptosis in RTCs. **(A)** Representative sections of nuclear DNA fragmentation staining were performed using TUNEL in different groups at 24, 48, and 72 h, respectively, after reperfusion. Scale bars = 50 µM. **(B)** Quantitative analysis of the number of TUNEL-positive RTCs. Data are presented as the mean ± SD (*n* = 5). ^∗^ *p* < 0.05, ^∗∗∗^ *p* < 0.001. The percentage of positive cells was analyzed with 5 individual magnification × 400 fields per group.

**FIGURE 6:**
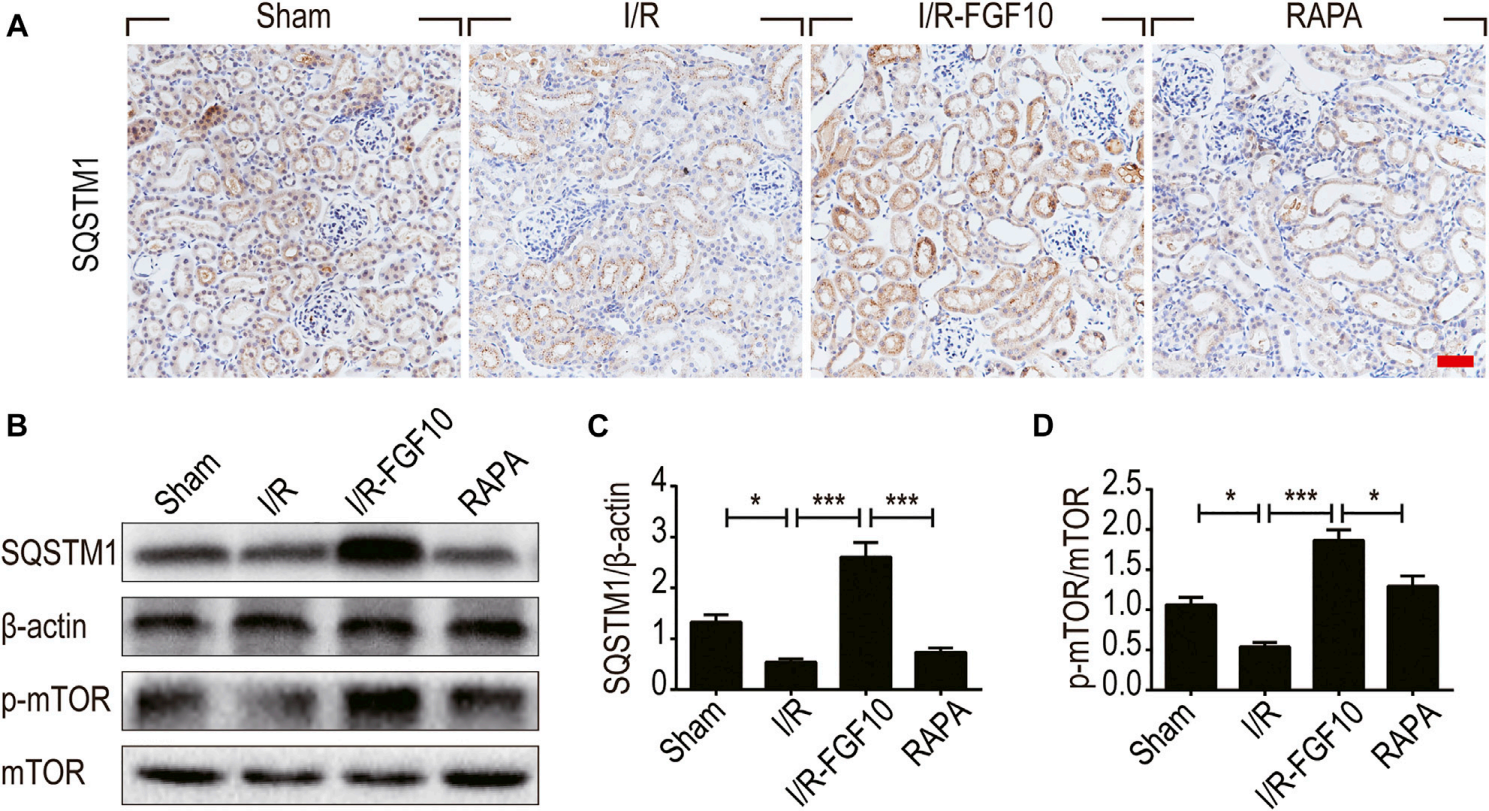
FGF10 increases the expression of SQSTM1 and p-mTOR in I/R rats. **(A)** IHC staining was performed at 2 days after reperfusion for SQSTM1 in kidney tissues from indicated animal groups. Scale bars = 50 µm. **(B)** The expression of SQSTM1, p-mTOR and mTOR were detected by western blotting (mean ± SEM; *n* = 5). β-actin was used as control. ^∗^ *p* < 0.05, ^∗∗∗^ *p* < 0.001. **(C,D)** Optical density analysis for SQSTM1 and p-mTOR, which were normalized to β-actin and mTOR, respectively.

**FIGURE 7:**
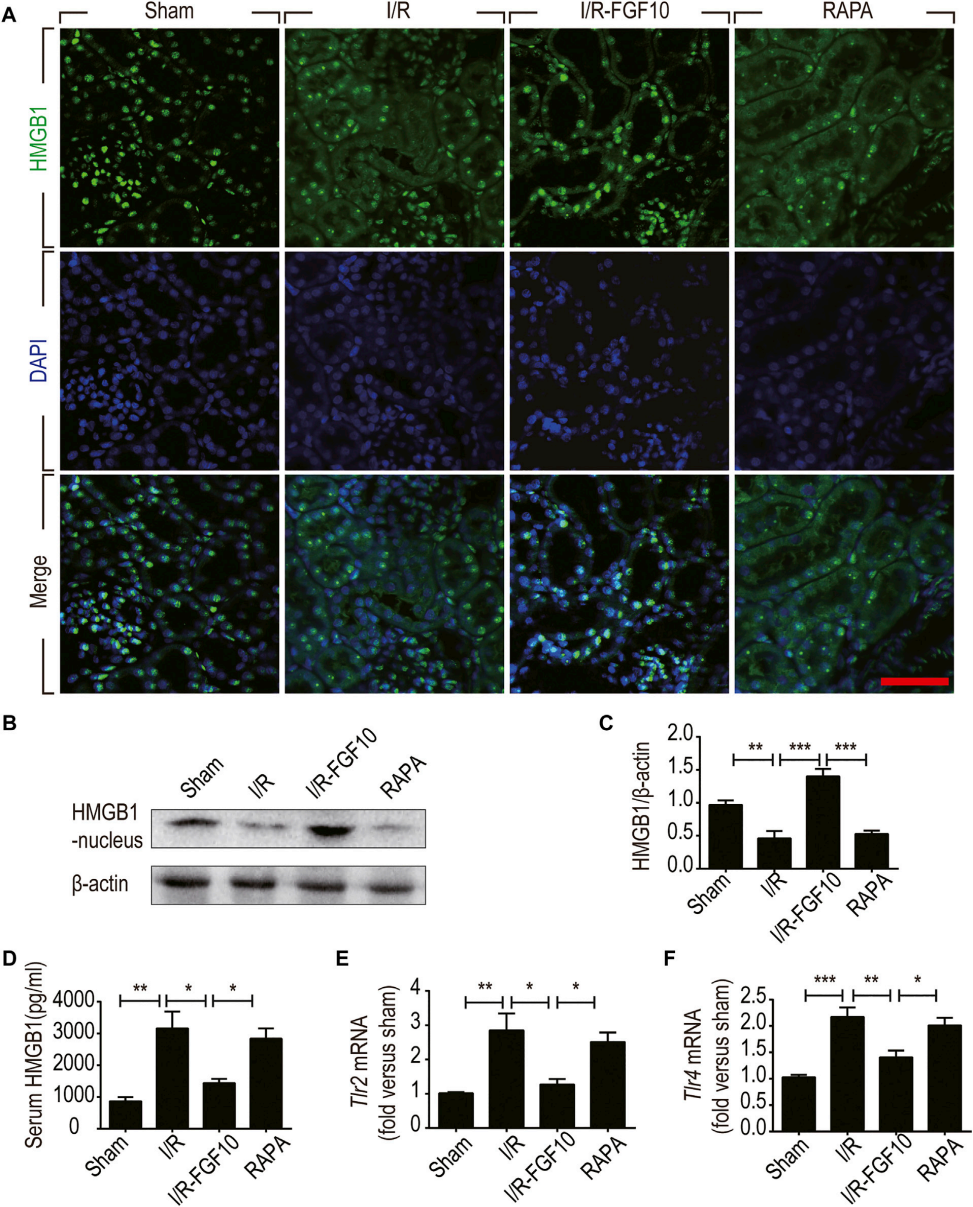
FGF10 inhibits the release of nuclear HMGB1 to the serum and regulates the TLR mRNA expression. **(A)** Immunofluorescence staining of HMGB1 at 2 days after reperfusion. Nuclei were labeled with DAPI (blue). Scale bars = 50 m. **(B,C)** Protein expression of HMGB1 in the nuclear fraction of renal tissues by Western blot and optical density analysis with β-actin as loading control (mean ± SEM; *n* = 5). ^∗∗^ *p* < 0.01, ^∗∗∗^ *p* < 0.001. **(D)** Level of serum HMGB1 was determined by ELISA (mean ± SEM; *n* = 5). ^∗^ *p* < 0.05, ^∗∗^ *p* < 0.01. **(E,F)** Expression of Tlr2 and Tlr4 mRNA in the kidney were examined by RT-qPCR and normalized to Gapdh. ∗*p* < 0.05, ∗∗*p* < 0.01, ∗∗∗*p* < 0.001.

The authors deeply apologize for these errors and state that these corrections do not change the scientific conclusions of the article in any way. The original article has been updated.

